# Progression and regression of incident cervical HPV 6, 11, 16 and 18 infections in young women

**DOI:** 10.1186/1750-9378-2-15

**Published:** 2007-07-12

**Authors:** Ralph P Insinga, Erik J Dasbach, Elamin H Elbasha, Kai-Li Liaw, Eliav Barr

**Affiliations:** 1 Department of Health Economic Statistics, Merck & Co., Inc., UG1C-60, P.O. Box 1000, North Wales, PA 19454-1099, USA; 2 Department of Health Economic Statistics, Merck & Co., Inc., UG1C-60, P.O. Box 1000, North Wales, PA 19454-1099, USA; 3 Department of Epidemiology, Merck & Co., Inc., UG1D-60, P.O. Box 1000, North Wales, PA 19454-1099, USA; 4 Department of Clinical Research, Merck & Co., Inc., UG3CD-28, P.O. Box 1000, North Wales, PA 19454-1099, USA

## Abstract

**Background:**

We describe type-specific progression, regression and persistence of incident human papillomavirus (HPV)-6-11-16 and -18 infections, along with type distribution in cervical intra-epithelial neoplasia (CIN) lesions.

**Methods:**

The study population consisted of 16–23 year-old women undergoing Pap testing and cervical swab polymerase chain reaction testing for HPV DNA at approximate 6 month intervals for up to 4 years in the placebo arm of a clinical trial of an HPV 16-vaccine. HPV types in incident infections were correlated with types in lesion biopsy specimens.

**Results:**

56.7% of CIN-1 and nearly one-third of CIN-2/3 lesions following incident HPV-6-11-16 or -18 infections did not correlate with the incident infection HPV type. Cumulative 36-month progression rates to CIN-2/3 testing positive for the relevant HPV type were highest for HPV-16 infections (16.5%), followed by HPV-18 (8.2%). Overall, 26.0% of CIN-1, 50.0% of CIN-2 and 70.6% of CIN-3 biopsies tested positive for HPV-6-11-16-18 infections.

**Conclusion:**

Women with a given HPV type may often be co-infected or subsequently infected with other types which may lead to subsequent cervical lesions. This issue has been addressed in this study reporting data for the natural history of HPV-6-11-16 and -18 infections and is a relevant consideration in designing future studies to evaluate the incidence/risk of CIN following other type-specific HPV infections.

## Background

Cervical cancer is the second most common cancer in women worldwide[1] and is caused by human papillomavirus (HPV) infection[2]. Organized screening using the Pap test has reduced cervical cancer rates in industrialized countries[3]. Because Pap testing does not address the root cause of cervical cancer (i.e., HPV infection), screening shifts the clinical burden of HPV disease from treating cancers to managing a large number of precancerous lesions[4,5]. It has been estimated that approximately 400,000 new cases of cervical intraepithelial neoplasia (CIN) are diagnosed in the United States each year, with annual costs of approximately $2.3 billion for cervical cancer screening and $700 million for CIN treatment[6]. The diagnosis and treatment of CIN can lead to anxiety concerning cancer risk and sexual functioning[7,8].

Prophylactic vaccines targeting HPV 6/11/16/18[9] and HPV 16/18 infections[10] have recently been shown to reduce the incidence of CIN due to these types. Policymakers worldwide are also evaluating technologies designed to enhance the diagnosis of CIN such as HPV tests and liquid-based cytology[11]. Data on the natural history of HPV infection are therefore critical for the evaluation of these and other emerging technologies.

The present study describes the type-specific progression and regression of incident HPV 6, 11, 16 and 18 infections, along with the observed distribution of these HPV types in CIN grade 1–3 lesions. A unique feature of the present study with respect to prior analyses of HPV infection natural history [12-15] is the correlation of HPV type observed in the incident infection with that detected in cervical lesion tissue specimens. Although the detection of CIN-1 lesions following HPV infection have received less attention than CIN-2/3 due to their lower oncogenic potential, a recent US study reported that more than half of women with incident CIN-1 were treated in clinical practice[16]. Policy models of HPV disease and costs have thus typically considered sub-clinical HPV infections (without clinically detectable CIN-1) as a distinct health state from progression to clinically detectable CIN-1 and we follow this dichotomy in the present study[17,18]. In this regard, a study of women with active HPV 6, 11, 16 and 18 infections undergoing colposcopy found that CIN-1-3 lesions were detected in 10%, suggesting that most HPV infections are not synonymous with clinically detectable CIN[19].

## Methods

### Study participants and procedures

The current evaluation focuses on women enrolled in the placebo arm of a randomized double-blind clinical trial of an HPV-16 vaccine (Merck Research Laboratories). The study population and trial design have been described elsewhere [19-22]. Briefly, the population consisted of 2,391 US women who on day 1 of the trial were 16–23 years of age, and did not report a history of pregnancy, abnormal Pap tests or more than 5 lifetime male sex partners. At enrollment, women provided written informed consent. The study was conducted in conformance with applicable federal and local requirements regarding ethical committee review and protection of human subjects participating in biomedical research. Women in the placebo arm received intramuscular injections visually indistinguishable from vaccine.

Women underwent type-specific endo/ecto-cervical swab HPV polymerase chain reaction (PCR) testing for HPV 6, 11, 16 and 18 infections at approximate 6 month intervals through 48 months of follow-up. The HPV testing methods utilized have been described in detail elsewhere [21,22]. Briefly, cervical swabs were prepared for PCR using a QIAamp DNA Blood kit (QIAGEN, Inc., Valencia, CA). DNA was analyzed by qualitative PCR using HPV 6, 11, 16 and 18 type-specific and gene-specific primers based on the L1, E6 and E7 genes for these types[20]. Beta-globin PCR assay was performed to verify that purified samples contained a sufficient quality and quantity of DNA for PCR amplification. PCR products were dot-blotted, hybridized to the corresponding ^32^P-labeled β-globin or HPV 6/11/16/18 gene-specific oligonucleotide, and visualized by autoradiography. Appropriate negative and positive controls were run with each assay and any specimen testing positive for at least 2 of the 3 genes was considered positive. Specimens testing positive for only 1 gene were considered positive if, on retesting, they were positive for 2 or 3 genes or the same single gene. Laboratory validation studies rigorously evaluated assay sensitivity against known copy number type- and gene-specific plasmids. The assay was shown to have a greater than 95% probability of detecting at least 13 copies per sample, with 95% upper confidence bounds for sample false negativity and false positivity of 0.7% and 0.8% respectively. All PCR assays were performed at Merck Research Laboratories (West Point, PA).

At visits where swab samples were obtained, cervical samples were also collected for liquid-based cytology (ThinPrep™, Cytyc) testing. An algorithm was used to guide further evaluation for cytologic findings. A single Pap test result of high-grade squamous intraepithelial lesion (HSIL), or repeated Pap tests showing low-grade lesions (LSIL) or atypical squamous cells of undetermined significance (ASCUS), prompted colposcopy. Investigators were allowed to manage single ASC-US and LSIL results based on local standards of care. All women attending the month 48 trial visit were referred for colposcopy, with biopsy performed if a CIN lesion was suspected. Cervical biopsy specimens were processed and assigned histologic diagnoses for purposes of medical management by central laboratory pathologists, and were typed by PCR for HPV 6, 11, 16 and 18 [20-22]. A multiplex PCR assay was used for HPV typing of biopsy specimens. This assay has previously been shown to be more sensitive than the Roche (Alameda, CA) reverse line blot PCR assay[23] with a limit of detection for HPV DNA of 6.1, 7.7, 5.5 and 6.5 copies per test for HPV 6, 11, 16 and 18 respectively[24]. The detection limit was defined as the lowest HPV copy number for which the one-sided lower 95% confidence bound on the probability of a positive PCR result exceeded 95%.

### Measures

**Figure 1:**
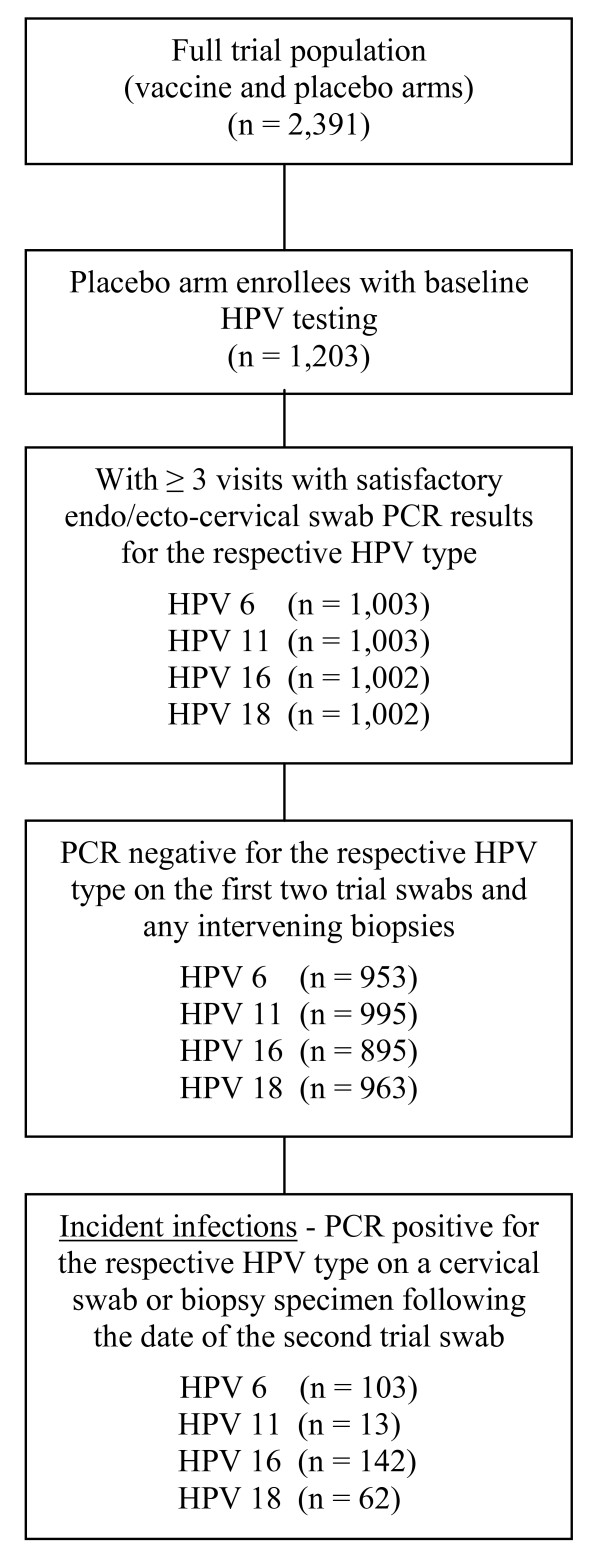
**Sample selection criteria for analyses of incident HPV 6, 11, 16 and 18 infections**. The final samples of women with incident HPV infections eligible for each type-specific analysis are represented within the bottom box, labeled by HPV type. These were comprised of placebo arm women with incident type-specific infections who were negative on PCR testing for the relevant HPV type on the first two trial endo/ecto-cervical swabs and any intervening cervical biopsies.

Incident HPV 6, 11, 16 and 18 infections were identified on cervical swab or biopsy specimens among woman negative for the relevant HPV type on their first 2 study swabs and any cervical biopsy specimens obtained on or prior to the second swab. Routine cervical swab HPV testing occurred at discrete intervals within the trial (approximately every 6 months). It is likely that initial positive HPV test results observed on a particular date reflected HPV infections that originated at some indeterminate time point between the last negative and initial positive test results. Consistent with our and others' earlier work,[19,25,26] we therefore assumed HPV infections occurred at the mid-point between the initial positive test date and the previous negative test, (please see figure 1).

Incident HPV infections were examined until either the detection of a CIN-1-3 lesion for which the cervical biopsy specimen tested PCR positive for the relevant HPV type ("progression"), or a negative cervical swab HPV test result(s) for that type ("regression"). Because women were required to have two consecutive negative swabs for a given HPV type prior to the start of an incident infection, if two consecutive negative swabs for that type were subsequently observed, the infection was assumed to have cleared at the midpoint in time between the final positive test and first negative swab result. However, there were also women with positive swab sample(s) for a given type followed by only a single negative swab prior to the trial concluding or their withdrawal. Among women with incident HPV-6 infections, 19 (18.4%) fell into this category, compared to 2 (15.4%), 16 (11.3%) and 9 (14.5%) with HPV-11, 16 and 18.

Since these women lacked two consecutive negative swabs, some previous analyses have evaluated them as censored at the date of the negative swab[27]. However, this would be expected to considerably under-estimate the actual regression rate of infection for these women. In actuality many would likely have cleared their infection around the time of their last positive swab and differ with respect to their subsequent course from women who were otherwise censored with a positive swab at their last trial visit. For instance, among 63 women with an incident HPV-6 infection followed by a negative swab sample, and at least one additional study swab, only 2 (3.2%) had a non-negative HPV-6 result on the second swab or a concurrent biopsy specimen. Similarly, for HPV-11, -16 and -18 infections the proportions were 0/6 (0.0%), 8/76 (10.5%) and 3/31 (9.7%). This would suggest that nearly all women with only a single negative HPV test at the conclusion of trial follow-up would have previously cleared their infections. We therefore evaluated these women as having cleared their infections at the midpoint in time between their final positive test and first negative swab result. Women observed to have positive swab or biopsy specimens, followed by a single negative swab, followed by a positive swab or biopsy specimen, were analyzed as having persistent infections. Women with a positive test on the date of their final trial swab sample were evaluated as censored.

From among all placebo-arm women diagnosed with CIN during the course of the trial, the prevalence of HPV 6, 11, 16 and/or 18 infections in CIN-1-3 lesions was estimated using PCR from the first biopsy diagnosis of CIN observed in the trial for each woman. Only the first CIN biopsy specimen was chosen for each woman to avoid selecting data for the same lesion multiple times in cases in which more than one biopsy specimen was taken during the course of follow-up. If multiple grades of CIN were observed for a woman among biopsies on a given date, the most severe histologic diagnosis was selected. HPV status for types other than 6, 11, 16 and 18 was unavailable.

Results were compared to data from previous US studies of the prevalence of type-specific HPV infections in biopsy-confirmed CIN lesions. Studies included performed PCR typing for multiple HPV types on cervical lesion tissue specimens. Analyses based on endo/ecto-cervical swab or lavage were excluded based on the increased potential for detecting HPV types unrelated to the cervical lesion of interest. To avoid double-counting in estimating the fraction of lesions attributable to individual or groups of HPV types, in instances where multiple HPV types were observed in a single lesion, the following procedure was undertaken. Each lesion testing positive for multiple HPV types was fractionally attributed to individual HPV types based on the relative proportion of lesions observed in each study to test positive for each single-type infection only, for that grade of lesion. For instance, if within a given study, a CIN-2/3 lesion tested positive for HPV types 6 and 16 and the number of single type-infected CIN-2/3 lesions for HPV-6 and -16 were 1 and 19 respectively, then (1/20) and (19/20) of the lesion was attributed to HPV-6 and -16.

### Statistical analysis

The cumulative proportion of HPV infections persisting without evidence of CIN, progressing to CIN 1, 2 and 3 and regressing, at 12, 24 and 36 months post-infection were estimated using Kaplan-Meier (K-M) methods[28]. Each outcome of persistent HPV infection was mutually exclusive. Thus, once an endpoint of progression to CIN 1, 2 or 3 was observed, a woman was no longer at risk for contributing towards the cumulative rate of regression, and vice versa. Ninety-five percent confidence intervals for cumulative proportions persisting, progressing and regressing were estimated through non-parametric bootstrapping of the K-M survivorship function with 1,000 replicates[29].

## Results

Among 1,203 women in the placebo arm of the trial who underwent cervical swab HPV PCR testing at enrollment, the prevalence of individual HPV types was as follows: HPV-6 (3.2%), HPV-11 (0.5%), HPV-16 (6.8%), HPV-18 (2.6%). The combined baseline prevalence of HPV 6, 11, 16 and 18 infections was 12.1%.

Characteristics of women with incident HPV-6 (n = 103), 11 (n = 13), 16 (n = 142) and 18 (n = 62) infections are presented in Table 1. Samples eligible for each type-specific analysis were generally similar. The median number of lifetime sexual partners at the time of incident HPV infection was five.

**Table 1:** Demographic and behavioral characteristics of eligible women with incident HPV 6, 11, 16 or 18 infections

Variable	HPV 6 N = 103 n (%)	HPV 11 N = 13 n (%)	HPV 16 N = 142 n (%)	HPV 18 N = 62 n (%)
*Age Group*				
16–19	6 (5.8)	0 (0.0)	13 (9.2)	8 (12.9)
20–23	77 (74.8)	7 (53.9)	95 (66.9)	43 (69.4)
24–27	20 (19.4)	6 (46.1)	34 (23.9)	11 (17.7)
*Race*				
Black	6 (5.8)	1 (7.7)	10 (7.0)	3 (4.8)
Hispanic	8 (7.8)	3 (23.1)	12 (8.5)	7 (11.3)
Other	4 (3.9)	1 (7.7)	8 (5.6)	2 (3.2)
White	85 (82.5)	8 (61.5)	112 (78.9)	50 (80.7)
*Smoking Status*				
Current Smoker	37 (35.9)	3 (23.1)	45 (31.7)	27 (43.6)
Ex-Smoker	18 (17.5)	4 (30.8)	28 (19.7)	9 (14.5)
Non-Smoker	48 (46.6)	6 (46.2)	69 (48.6)	26 (41.9)
*Age at first sexual intercourse*				
≤ 14	4 (3.9)	1 (7.7)	13 (9.2)	9 (14.5)
15–18	76 (73.9)	10 (76.9)	105 (73.9)	46 (74.2)
≥ 19	23 (22.3)	2 (15.4)	24 (16.9)	7 (11.3)
*Lifetime Number of Sexual Partners*				
1	7 (6.8)	1 (7.7)	12 (8.5)	2 (3.2)
2–3	23 (22.3)	5 (38.5)	24 (16.9)	11 (17.8)
4–6	47 (45.7)	7 (53.8)	73 (51.4)	35 (56.4)
7–9	21 (20.4)	0 (0.0)	27 (19.0)	10 (16.1)
10–14	5 (4.8)	0 (0.0)	6 (4.2)	4 (6.4)

HPV = human papillomavirus

Following the date of their incident HPV 6, 11, 16, and 18 infections, women averaged an additional 21.2, 16.1, 22.1 and 21.4 months of follow-up respectively. The number of CIN lesions testing positive or negative for the HPV type observed in the incident infection, during the subsequent course of follow-up, is reported in Table 2. Overall, more than half of CIN-1 lesions were negative for the HPV type observed in the incident infection, compared to nearly one-third of CIN-2/3 lesions. Only for analyses of CIN-2/3 lesions following incident HPV-16 infections was there a high type-specific concordance (85.0%). The average estimated time from onset of each type-specific infection to a CIN-1 (9.3 months) or CIN-2/3 (11.8 months) lesion positive for the same type was observed to be shorter than for instances where a CIN-1 (16.5 months) or CIN-2/3 lesion (17.2 months) was detected that was negative for the relevant type. However, there was also substantial overlap in times to CIN detection, with the interquartile range (25^th^–75^th ^percentile) of times to detection of type-concordant CIN-2/3 lesions covering the 10^th ^to 70^th ^percentiles of times to detection of type-discordant CIN-2/3.

**Table 2:** Number of incident HPV 6, 11, 16 and 18 infections with subsequent CIN lesions testing positive or negative for the HPV type observed in the incident infection

HPV Type	CIN 1-Positive for type (% of CIN1)	CIN 1-Negative for type* (% of CIN 1)	CIN 2/3-Positive for type (% of CIN 2/3)	CIN 2/3-Negative for type* (% of CIN 2/3)
HPV 6	14 (43.8%)	18 (56.2%)	2 (28.6%)	5 (71.4%)
HPV 11	0 (0.0%)	3 (100.0%)	0 (-)	0 (-)
HPV 16	23 (51.1%)	22 (48.9%)	17 (85.0%)	3 (15.0%)
HPV 18	5 (29.4%)	12 (70.6%)	4 (57.1%)	3 (42.9%)
Total	42 (43.3%)	55 (56.7%)	23 (67.6%)	11 (32.4%)

* Percentages negative for type refer to the proportion of CIN 1 and CIN 2/3 cases that tested negative for the HPV type listed in that row at left.

CIN = cervical intraepithelial neoplasia; HPV = human papillomavirus

The cumulative proportion of females with incident HPV 6, 11, 16 and 18 infections progressing to CIN-1-3 testing positive for the relevant HPV type on biopsy, persisting with type-specific infection in the absence of detected CIN, or regressing to negative for HPV infection for the relevant type on a cervical swab, at 12, 24 and 36 months of follow-up is presented in Table 3. Cumulative 36-month progression rates to CIN-1 ranged from 0.0% for HPV-11 to 20.7% for HPV-16. Progression rates over 36 months to CIN-2/3 were highest for HPV-16 infections (16.5%), followed by HPV-18 (8.2%). No women were diagnosed with invasive cervical cancer. By 36 months all HPV 6, 11 and 16 infections had either regressed, or progressed to CIN, with 8.2% of HPV-18 infections persisting. The cumulative regression rate was highest for HPV-11 (100.0%) and lowest for HPV-16 (62.8%).

**Table 3:** Type-specific persistence, regression and progression of incident HPV 6, 11, 16, 18 infections

	**Proportion Persisting Without CIN Due to Type (95% CI)**	**Proportion for Which Type Regressed (95% CI)**	**Proportion Progressed to CIN 1 Due to Type (95% CI)**	**Proportion Progressed to CIN 2 Due to Type (95% CI)**	**Proportion Progressed to CIN 3 Due To Type (95% CI)**
**HPV 6 **(n = 103)					
12 Months	26.0 (17.5, 35.0)	62.3 (52.9, 71.8)	9.6 (4.0, 15.5)	2.1 (0.0, 5.5)	0.0 (-)
24 Months	0.0 (-)	84.0 (75.5, 91.3)	13.9 (6.7, 21.1)	2.1 (0.0, 5.5)	0.0 (-)
36 Months	0.0 (-)	84.0 (75.5, 91.3)	13.9 (6.7, 21.1)	2.1 (0.0, 5.5)	0.0 (-)
**HPV 11 **(n = 13)					
12 Months	14.6 (0.0, 42.9)	85.4 (57.1, 100.0)	0.0 (-)	0.0 (-)	0.0 (-)
24 Months	0.0 (-)	100.0 (-)	0.0 (-)	0.0 (-)	0.0 (-)
36 Months	0.0 (-)	100.0 (-)	0.0 (-)	0.0 (-)	0.0 (-)
**HPV 16 **(n = 142)					
12 Months	47.2 (37.9, 56.6)	35.4 (27.3, 44.5)	10.5 (5.7, 16.5)	4.5 (1.0, 8.6)	2.4 (0.0, 5.5)
24 Months	15.8 (8.6, 24.8)	53.0 (43.5, 63.2)	18.3 (11.5, 26.0)	9.1 (3.8, 14.8)	3.8 (0.8, 8.2)
36 Months	0.0 (0.0, 7.1)	62.8 (50.0, 73.1)	20.7 (12.1, 29.6)	9.1 (3.8, 14.8)	7.4 (1.9, 14.0)
**HPV 18 **(n = 62)					
12 Months	52.9 (38.9, 67.1)	34.8 (22.3, 48.2)	6.8 (1.6, 13.7)	5.5 (0.0, 12.2)	0.0 (-)
24 Months	16.3 (5.3, 28.7)	66.0 (51.4, 79.0)	9.5 (1.9, 18.5)	5.5 (0.0, 12.2)	2.7 (0.0, 9.6)
36 Months	8.2 (0.0, 23.1)	74.1 (55.9, 89.5)	9.5 (1.9, 18.5)	5.5 (0.0, 12.2)	2.7 (0.0, 9.6)

CIN = cervical intraepithelial neoplasia; HPV = human papillomavirus; CI = confidence interval

Among all placebo arm women diagnosed with CIN (n = 267), regardless of HPV status, there were 442 CIN-1-3 cervical biopsy specimens. From the first CIN biopsy specimens obtained for these 267 women, 22 (8.2%) lacked HPV typing. This left an eligible sample of 245 initial CIN biopsies, of which 192 were CIN-1, 36 CIN-2 and 17 CIN-3. Overall, 26.0% of CIN-1, 50.0% of CIN-2 and 70.6% of CIN-3 biopsies tested PCR-positive for HPV 6, 11, 16 or 18 (Table 4). Of lesions testing positive for these types, 5 (4.9%) were observed to test positive for more than one of these types. No CIN cases tested positive for HPV-11. HPV-6, in the absence of HPV-16 and/or 18 co-infection, was observed in 5.2% of CIN-1, 5.6% of CIN-2 and 5.9% of CIN-3 cases.

**Table 4:** Prevalence of HPV 6, 11, 16 and 18 infections in CIN 1–3 lesions

**Histologic Diagnosis/HPV Type(s)**	**n (%)**
**CIN 1**	
HPV 6	10 (5.2)
HPV 6 and 16	1 (0.5)
HPV 6, 16 and 18	1 (0.5)
HPV 16	30 (15.6)
HPV 16 and 18	1 (0.5)
HPV 18	7 (3.6)
Negative for HPV 6, 11, 16 and 18	142 (74.0)
Total	192 (100.0)
**CIN 2**	
HPV 6	2 (5.6)
HPV 6 and 16	1 (2.8)
HPV 16	12 (33.3)
HPV 18	3 (8.3)
Negative for HPV 6, 11, 16 and 18	18 (50.0)
Total	36 (100.0)
**CIN 3**	
HPV 6	1 (5.9)
HPV 16	10 (58.8)
HPV 16 and 18	1 (5.9)
Negative for HPV 6, 11, 16 and 18	5 (29.4)
Total	17 (100.0)

HPV = human papillomavirus; CIN = cervical intraepithelial neoplasia

The overall estimated proportion of CIN-1 lesions attributable to HPV 6, 11, 16 and 18 infections (25.0%) in prior US studies (Table 5) was nearly identical to that observed in the present analysis (26.0%). For CIN-2/3, the estimated proportion of lesions attributable to these four types was also relatively similar between the present (56.6%) and prior (63.6%) studies. However, no prior studies documented the presence of HPV-6 in CIN-2/3 lesions. Only two of the seven prior studies, by Evans et al. (1 CIN-1) and Hu et al. (5 CIN-1, 14 CIN-2/3), reported lesions explicitly identified as containing multiple HPV types[30,31]. One additional US study of CIN-2/3 lesions was excluded from the analysis as 67% of lesions were reported as testing positive for multiple HPV types (41% double/26% triple infections), suggestive of potential problems with testing methods for the 3 HPV types examined[32].

**Table 5:** Percent of CIN cases estimated to be attributable to nine specific HPV types & any HPV type in prior US studies

				% of Cases Estimated to be Attributable to Specific HPV types									
					
Year	Author	N	Testing Method	HPV 6	HPV 11	HPV 16	HPV 18	HPV 31	HPV 33	HPV 45	HPV 52	HPV 58	Any HPV type*
**CIN 1 **^30,31,39–41,43^													
2006	Srodon, M	36	PCR/Biopsy	2.8	2.8	11.1	16.7	5.6	5.6	5.6	2.8	2.8	100.0
2005	Hu, L^†^	45	PCR/Biopsy			6.4	2.2	3.1	0.0	2.2	12.2	7.2	87.0
2002	Evans, M^†^	28	PCR/Biopsy	4.5	3.6	7.1	3.6	7.1	3.6	3.6	7.1	0.0	100.0
1998	Aoyama, C	11	PCR/Biopsy	0.0	0.0	27.3	9.1	9.1	9.1				54.5
1998	Quade, B	30	PCR/Biopsy	3.3	3.3	10.0	3.3	6.7	0.0	0.0	0.0	0.0	73.3
1996	Isacson, C	40	PCR/Biopsy	5.0	10.0								

Total		190		3.6	4.8	9.9	6.7	5.6	2.7	2.9	6.1	3.0	87.4
**CIN 2/3 **^30,31,39–43^													
2006	Srodon, M	116	PCR/Biopsy	0.0	0.0	75.0	4.3	5.2	1.7	0.0	0.9	3.4	100.0
2005	Hu, L^†^	97	PCR/Biopsy			38.9	9.6	11.6	1.0	1.1	5.0	2.6	93.6
2002	Evans, M	22	PCR/Biopsy	0.0	0.0	68.2	4.5	9.1	4.5	4.5	0.0	4.5	100.0
1998	Aoyama, C	21	PCR/Biopsy	0.0	0.0	52.4	0.0	19.0	19.0				95.2
1998	Quade, B	19	PCR/Biopsy	0.0	0.0	52.6	5.3	0.0	15.8	0.0	0.0	0.0	78.9
1996	Isacson, C	11	PCR/Biopsy	0.0	0.0	45.5	0.0	18.2	9.1	0.0	0.0	0.0	100.0
1993	Shroyer, K	13	PCR/Biopsy	0.0	0.0	61.5	0.0						92.3

Total		299		0.0	0.0	58.1	5.5	8.8	4.2	0.8	2.2	2.8	95.9

* As determined by consensus PCR primers sensitive to positivity for at least 10 HPV types.

^† ^Lesions in these studies containing multiple HPV types were fractionally attributed to individual HPV types as described in the Methods section. Without this method of attribution, original figures for HPV prevalence differing from those reported in the table were as follows: Evans et al., CIN 1 (HPV 6 – 7.1%); Hu et al., CIN 1 (HPV 16 – 8.9%, 31 – 6.7%, 52 – 13.3%, 58 – 11.1%), CIN 2/3 (HPV 16 – 40.2%, 18 – 12.4%, 31 – 13.4%, 45 – 2.1%, 52 – 8.2%, 58 – 6.2%)

CIN = cervical intraepithelial neoplasia; HPV = human papillomavirus; PCR = polymerase chain reaction

## Discussion

This analysis has described type-specific progression, persistence and regression of incident HPV 6, 11, 16 and 18 infections, along with the observed distribution of these HPV types in CIN-1-3 lesions. To our knowledge, this is the first analysis to examine the type-specific detection of histologically confirmed CIN-1 following incident HPV infections, and to correlate the HPV type observed in incident HPV infections with that subsequently detected in cervical lesion tissue specimens in estimating progression rates. The perspective adopted is particularly useful for informing HPV disease natural history models used in policy evaluations of emerging technologies[17,18,33].

A salient inference from this analysis is that women infected with a given HPV type are often co-infected or subsequently infected with other HPV types which may be responsible for subsequent cervical lesions. This was especially true for CIN-1, where the majority of detected lesions following incident HPV 6, 11, 16 or 18 infections did not correlate with the incident infection HPV type, however was also observed for nearly one-third of CIN-2/3 cases.

Prior studies of the natural history of incident HPV infections have generally not performed HPV typing of CIN lesion tissue specimens and have thus estimated results based on all lesions observed, regardless of HPV type, following detection of a given infection[12,15]. This perspective can be clinically useful for assessing the overall risk of being subsequently diagnosed with cervical neoplasia for women with HPV infections of a specific type. For instance, Koutsky et al. reported a 24 month risk of CIN-2/3 following incident HPV-6 or -11 infection of 26%[13]. This compares to a figure of 1.9% observed over the same time period in the present study, based on HPV typing of lesion tissue specimens. Thus it is possible that women with incident HPV-6 and -11 infections are clinically at relatively high-risk for CIN-2/3 compared to other women,[15] but that in the majority of cases, the lesions are caused by other HPV types. In general, the exclusion of disease testing positive for HPV types discordant from the incident infection would be expected to yield lower estimates of CIN risk following type-specific infection in the present study, compared to previous analyses. Correlation of HPV typing between infections and lesions can be particularly helpful for modeling studies evaluating HPV vaccines and tests[17,18], where risks associated with individual HPV types or groups of types are typically of interest.

The reporting of data for clinically detected CIN-1 as a distinct endpoint represents a second difference in analytic method compared to most prior HPV natural history studies, which have focused upon CIN-2/3 development [13-15]. Although CIN-2/3 represents a more proximate precursor to cervical cancer, CIN-1 is also often treated in clinical practice in the US, ^16^and results in substantial healthcare costs[6].

In the present study, the initial biopsy result showing evidence of CIN was used for the purposes of denoting progression of infection to clinically detected CIN-1. These women were not eligible to further contribute to the endpoints of CIN-2 or -3 should they have further progressed, and some women with CIN-1 were subsequently treated, thereby preventing progression. In most recent HPV natural history models[17,18], individual transition probabilities have been utilized to model rapid progression from HPV infection directly to CIN-2 or -3, more deliberate progression of infection to CIN-1, and regression of HPV infection. Additional health state transitions have been used to model progression from CIN-1 to CIN-2, and regression of CIN-1. Data in this study were analyzed in a manner to inform each of the described transitions originating from HPV infection. Other studies describing progression and regression rates for CIN-1 have been reviewed elsewhere [34-36].

Based on this perspective, risks of progression of incident HPV infection to CIN-2/3 from the present analysis are not directly comparable to those reported in prior natural history studies where CIN-1 was not evaluated as a separate endpoint [13-15]. All else equal, relatively lower risks of progression to CIN-2/3 over time would be expected in the present study, as cases of CIN-1 that otherwise may have progressed to CIN-2/3, and been counted as CIN-2/3 endpoints in prior analyses, were truncated at the point of the initial CIN-1 diagnosis. Indeed, a study by Winer et al. reported a 36 month risk of progression of HPV-16 and -18 infections to CIN-2/3 (regardless of HPV type) of 27.2%, well above the 14.0% figure estimated in the present analysis[14].

However, a common finding across studies has been the relatively high risk of progression of HPV-16 and -18 to cervical neoplasia compared to other HPV types [13-15], with nearly one-third of women newly infected with these types subsequently diagnosed with CIN in the present study. Approximately 85% of HPV-6 and -11 infections were observed to regress without resulting in a CIN diagnosis, however these HPV types cause 90–100% of condyloma acuminata, the classic form of genital warts[37,38].

This study adds HPV typing of 192 CIN-1 and 53 CIN-2/3 lesion tissue specimens to previous US data for 190 CIN-1 and 299 CIN-2/3 lesions respectively[30,31,39-43]. The distribution of HPV types 6, 11, 16 and 18 in cervical lesions was generally similar to that observed in aggregate across prior studies. However, a small proportion of CIN-2/3 lesions also tested positive for HPV-6 alone (5.7%) or in combination with HPV-16 (1.9%). Since testing was not conducted for types other than HPV 6, 11, 16 and 18 it is possible that other types were also present in combination with HPV-6. No CIN-2/3 lesions positive for HPV-6 have been reported in prior US studies, however a large US study of nearly 400 invasive cervical cancer tissue specimens by Schwartz et al. revealed a prevalence of HPV-6 alone in 1.8% of cases, and HPV-6 in combination with other types in 3.1% of cases[44]. The presence of HPV-6 in CIN-2/3 lesions has not been examined in prior international reviews and represents an area for further research[45].

## Limitations

A limitation of this study is that although liquid-based cytology was conducted at 6 month intervals, with rigorous follow-up of suspected abnormalities, and women were referred for colposcopy at the conclusion of the study, the detection of some CIN lesions may have been missed or delayed. Thus, progression rates to CIN may be conservative. A prior study of HPV natural history by Winer et al. performed colposcopy at regularly scheduled visits on all women and reported relatively higher risks of progression to CIN-2/3[14]. However, as described previously, because HPV typing was not conducted for lesion tissue specimens, and progression over time to CIN-1 was not separately evaluated, all else equal, the risk of progression of specific HPV types to CIN-2/3 would be expected to be somewhat liberal for disease modeling purposes. In the absence of a single study overcoming the limitations of both analyses, in our own HPV infection modeling work[46], we have chosen to average estimates obtained from the Winer and present study to account for the divergent effects of these limitations.

Also, as previously noted, testing data were not available in this study population for additional HPV types beyond HPV 6, 11, 16 and 18. Thus, it was not possible to fully characterize multi-type HPV infections in lesions where present or explore the natural histories of other HPV types.

Finally, women in the clinical trial were required to have 5 or fewer lifetime sexual partners at enrollment. In the sub-sample of women examined in this analysis that developed incident HPV 6, 11, 16 or 18 infections over the course of follow-up, the median number of lifetime sexual partners at the time of infection was five. There are not published nationally representative US data on the lifetime number of sexual partners among women developing incident HPV infections from which to make comparisons of external generalizability. However, in a further investigation of this potential limitation, we found through Cox regression analysis that the risk of developing CIN among women infected with HPV 6, 11, 16 or 18 did not vary by the lifetime number of sexual partners at the time of infection (hazard ratio = 1.04; 95% CI 0.98–1.11).

## Conclusion

Over the past two decades there has been substantial research demonstrating the causal link between HPV infection and cervical neoplasia[47], and the relatively high proportion of cervical cancers associated with certain HPV types, particularly types 16 and 18[48]. With the arrival of new technologies, such as HPV tests and vaccines targeting specific HPV types, there has also arisen a need to accurately model the natural history of infection for individual or groups of HPV types for policy evaluations[9,10,17,18]. Epidemiologic studies to date have generally not been specifically designed to evaluate HPV infection or disease natural history in a manner ideal for infectious disease modeling. This study has contributed additional data for modeling outcomes of incident HPV 6, 11, 16 and 18 infections. However, further studies with rigorous disease ascertainment would be helpful, particularly for incident infections caused by other HPV types, and the progression and regression of incident cervical neoplasia caused by specific HPV types.

## Competing interests

This study was supported by Merck Research Laboratories, where the authors are currently employed. Authors potentially hold stock and/or stock options in the company. Merck & Co., Inc. has developed a quadrivalent HPV vaccine.

## Authors' contributions

All authors participated in the design of the study and drafting of the manuscript, and have read and approved the final version. RI conceived of the study and performed the data and statistical analyses.
